# Differential Effects of Drug Interventions and Dietary Lifestyle in Developing Type 2 Diabetes and Complications: A Systems Biology Analysis in LDLr−/− Mice

**DOI:** 10.1371/journal.pone.0056122

**Published:** 2013-02-15

**Authors:** Marijana Radonjic, Peter Y. Wielinga, Suzan Wopereis, Thomas Kelder, Varshna S. Goelela, Lars Verschuren, Karin Toet, Wim van Duyvenvoorde, Bianca van der Werff van der Vat, Johanna H. M. Stroeve, Nicole Cnubben, Teake Kooistra, Ben van Ommen, Robert Kleemann

**Affiliations:** 1 Microbiology and Systems Biology, TNO, Zeist, The Netherlands; 2 Metabolic Health Research, TNO, Leiden, The Netherlands; 3 Quality and Safety, TNO, Zeist, The Netherlands; 4 Pharmacokinetics and Human Studies, TNO, Zeist, The Netherlands; University of Tor Vergata, Italy

## Abstract

Excess caloric intake leads to metabolic overload and is associated with development of type 2 diabetes (T2DM). Current disease management concentrates on risk factors of the disease such as blood glucose, however with limited success. We hypothesize that normalizing blood glucose levels by itself is insufficient to reduce the development of T2DM and complications, and that removal of the metabolic overload with dietary interventions may be more efficacious. We explored the efficacy and systems effects of pharmaceutical interventions versus dietary lifestyle intervention (DLI) in developing T2DM and complications. To mimic the situation in humans, high fat diet (HFD)-fed LDLr−/− mice with already established disease phenotype were treated with ten different drugs mixed into HFD or subjected to DLI (switch to low-fat chow), for 7 weeks. Interventions were compared to untreated reference mice kept on HFD or chow only. Although most of the drugs improved HFD-induced hyperglycemia, drugs only partially affected other risk factors and also had limited effect on disease progression towards microalbuminuria, hepatosteatosis and atherosclerosis. By contrast, DLI normalized T2DM risk factors, fully reversed hepatosteatosis and microalbuminuria, and tended to attenuate atherogenesis. The comprehensive beneficial effect of DLI was reflected by normalized metabolite profiles in plasma and liver. Analysis of disease pathways in liver confirmed reversion of the metabolic distortions with DLI. This study demonstrates that the pathogenesis of T2DM towards complications is reversible with DLI and highlights the differential effects of current pharmacotherapies and their limitation to resolve the disease.

## Introduction

Type 2 diabetes mellitus (T2DM) is a multifactorial metabolic disease that is associated with multiple life-threatening complications such as cardiovascular disease (CVD) [1]. 366 million people suffer from diabetes worldwide, with another 280 million at high risk of developing the disease [2]. Despite major advances in understanding the pathogenesis of the disease and effective therapies to normalize plasma glucose and to reduce risk factors, incident rates of T2DM steadily increase and cardiovascular complications remain the largest cause of morbidity and mortality in T2DM patients [3]. Also, therapeutic treatment of other important comorbidities, including nephropathy, retinopathy and non-alcoholic liver disease, remain challenging. There is increasing evidence that current T2DM disease management concentrates on a symptom of the disease, i.e. blood glucose, but leaves the true underlying cause, viz. metabolic overload, unaffected, and thus fails to impact upon the major complications associated with T2DM [4]–[6]. It is assumed that diet-related changes in lifestyle may reverse metabolic distortions in central organs (and systemically), thereby exerting a beneficial effect on causal paths of the disease.

Here we sought further evidence for the hypothesis that normalizing blood glucose levels by itself is insufficient to reduce the development of complications, and that removal of the metabolic overload with dietary interventions may be more efficacious. These hypotheses were tested in a diet-inducible experimental model of disease, high-fat fed low-density lipoprotein receptor-deficient (LDLr−/−) mice which develop multiple T2DM-related complications in liver, aorta and kidney. Three types of pharmacological interventions were tested, each of which representing a specific category of drugs. The first category encompassed conventional anti-diabetic drugs [7], viz. metformin, a sulfonylurea compound (glibenclamide), two thiazolidinediones (rosiglitazone and pioglitazone) and a DPP-4 inhibitor (sitagliptin). The second category contained lipid-modulating compounds (fenofibrate, the LXR agonist T0901317 and atorvastatin) which also possess anti-inflammatory vasculoprotective properties [8]. Thirdly, we evaluated two anti-inflammatory compounds (rofecoxib and salicylate) with the aim to interfere in metabolic inflammation, a driver of the pathogenesis towards complications [9], [10]. Finally and different from the drug approach, we explored the effects of a specific lifestyle intervention, viz. reduction of the metabolic pressure by switching to a low-fat chow diet.

To mimic the human disease situation, drug and lifestyle interventions were started once early hallmarks of the disease including central adiposity, hyperglycemia, hyperinsulinemia and dyslipidemia were established. The different treatment strategies were evaluated with respect to plasma risk factors (glucose, insulin, triglycerides and cholesterol), diabetic complications (fatty liver, microalbuminuria, atherosclerosis) as well as metabolic disturbances assessed by changes in metabolite, protein and gene expression profiles in liver and plasma. The results may provide insight into the shortcomings of current T2DM treatment regimens, but could also open new avenues for novel therapeutic paradigms based on a systems approach.

## Materials and Methods

### Animals Experiment and Interventions

Animal experiments were approved by an independent Committee on the Ethics of Animal Experiments (Zeist, The Netherlands) (Permit Number: 2935). LDLr−/− mice had free access to low fat maintenance chow diet (Sniff R/M diet V1530, Uden, The Netherlands) until the start of the study. N = 132 animals were fed a high fat diet (HFD) for nine weeks to established obesity-associated hyperglycemia, hyperinsulinemia, hypertriglyceridemia and hypercholesterolemia. Separate animals (n = 9) remained on chow for the entire experiment (reference age-matched control). The HFD-fed mice were matched into thirteen groups based on body weight. The first group (n = 9) was sacrificed immediately to define the condition at the start of the interventions with drugs and lifestyle. The second group (n = 15) was continued on HFD and remained untreated. The pharmacological intervention groups (each n = 9) received HFD supplemented with one of the following drugs (all w/w): the anti- diabetic drug metformin (0.250%), glibenclamide (0.010%), sitagliptin (0.020%), rosiglitazone (0.010%) and pioglitazone (0.010%); the lipid modulating compounds fenofibrate (0.050%), T0901317 (0.010%) and atorvastatin (0.010%); the anti-inflammatory compounds salicylate (0.40%) and rofecoxib (0.034%). The last intervention group (n = 9) was switched back to chow (dietary lifestyle intervention; DLI). Spot urine and 5 hr fasting blood samples were collected over time and after 7 weeks of treatment, mice were sacrificed to analyze organs.

### Analyses in Plasma and Urine

Plasma lipids were measured with kits 11489437 (cholesterol) and 11488872 (triglycerides) from Roche Diagnostics (Almere, The Netherlands) [11]. Plasma glucose was quantified by glucose hexokinase method (Instruchemie, Delfzijl, The Netherlands) and plasma insulin by ELISA (Ultrasensitive mouse insulin ELISA, Mercodia, Uppsala, Sweden) to calculate QUICKI insulin sensitive index as reported [12]. For analysis of cytokines and hormones, Bio-Rad 23-multiplex (#171-F11241) and Bio-Plex_Pro-Mouse diabetes 8-plex (#171-F7001M) were used, respectively. To assess glomerular barrier function, urinary albumin (Exocell Inc. Philadelphia, PA, USA) and creatinine concentrations were determined (Bethyl Laboratories Inc. Montgomery, TX, USA).

### Analysis of Atherosclerosis and Hepatosteatosis

Atherosclerosis was analyzed blindly in hematoxylin-phloxine-saffron-stained serial cross-sections (n = 4 per mouse) of the aortic arch (40 µm intervals) and scored essentially as described [13] using an Olympus BX51 microscope and CellˆD software (Olympus, Zoeterwoude, The Netherlands).

Liver homogenates were prepared to extract lipids to quantify steatosis by the Bligh-and-Dyer method using silica-gel-60 plates and thin layer chromatography. A Hewlett Packard Scanjet 4500c with Tina software (version-2.09) was used to integrate density areas and calculate lipid concentrations.

### Hepatic Transcriptome Analysis

Total RNA was isolated using the NucleoSpin® RNA II kit from Macherey-Nagel. The quality control of RNA samples, RNA labelling and hybridisation were performed at ServiceXS (Leiden, The Netherlands) as previously described [14]. Per sample, 750 ng of cRNA was used to hybridise to the MouseRef-8 v2 Expression BeadChip (Illumina, Inc., San Diego, CA, U.S.A.). Image analysis and extraction of expression data were performed with Illumina Genomestudio Gene Expression software using default settings. The microarray data from this publication have been submitted to the Array Express repository (accession number E-MTAB-1063) and will be additionally available via Phenotype database http://ls10ds.dbnp.org.

### Microarray Data Preprocessing, Visualization, Statistical and Pathway Analysis

The probe-level, background subtracted expression values were used as input for lumi package [15] of the R/Bioconductor (http://www.bioconductor.org; http://www.r-project.org) to perform quality control and a quantile normalization. Unexpressed probes (p>0.01 in all experiments) were removed from the further analyses, leaving 15725 probes for the analysis. Differentially expressed probes were identified using the limma package of the R/Bioconductor [16]. The calculated P-values were corrected for multiple testing. False discovery rate of 5% (q-value<0.05) was used as a threshold for significance of the differential expression.

Hierarchical clustering of differentially expressed transcripts and metabolites and proteins with significantly different concentrations were performed in R (http://www.r-project.org), using Pearson correlation to calculate the distance matrix and complete linkage for hierarchical clustering. Identification of overrepresented Gene Ontology functional categories among differentially expressed probes was performed using MetaCore (GeneGo Inc.) and DAVID Functional Annotation Clustering tool (version date February 2012) [17]. Heatmaps representing P-values of the selected functional categories across the treatment groups and gene expression of selected genes was generated using HeatmapViewer module within the GenePattern analysis suite [18]. The network analysis of the differentially expressed probes in the lifestyle group was performed using Ingenuity Pathways Analysis (Ingenuity Systems. Available: www.ingenuity.com. Accessed 2012).

### GC-MS Analysis of a Broad Range of Metabolites in Plasma and Liver

The GC-MS method used for the measurement of a broad range of metabolites was reported previously [19]. In the present study, 20 µL plasma or 10 mg lyophilized liver tissue were extracted and further derivatized. For liver, a 30 minute ultrasonic extraction was performed. The protocols for control of performance of the GC-MS system, normalization of study samples and correction of systematic errors and annotation of metabolites have been reported in [20]. For quality control commercially available mouse plasma and liver biopsies from LDLr−/− mice were used.

### Liver Extraction and SPE LC-MS/MS Analysis of Eicosanoids

Liver samples were collected and directly supplemented with an inhibitor cocktail containing paraoxon, BHT, AUDA, indomethacin, and PMSF to prevent eicosanoid oxidation and breakdown. The liver samples were precipitated with methanol (1∶5), and incubated for 30 minutes on ice. Samples were subsequently centrifuged (5′ at 3000×*g* and 4°C) and the supernatant was transferred to a glass tube. Just before loading on activated HLB columns, 4.75 mL MQ water containing 0.1% v/v FA was added to the methanol extract, diluting the extract to 20% methanol. After loading, the columns were washed with 2 mL 20% methanol in MQ water containing 0.1% FA, and the columns were allowed to dry for 15 minutes. The SPE columns were eluted with 2 mL methanol and the samples were captured in tubes already containing 20 µL of 10% glycerol and 500 µM BHT in ethanol. The tubes were placed in a water bath at 40°C and the methanol was evaporated under a gentle stream of nitrogen, reconstituted in 100 µl ethanol containing another internal standard (CUDA) and immediately used for LC-MS/MS analysis as described by Balvers et al. [21].

### Biological Interpretation of Hepatic Metabolome Data

Functional analysis of the data was performed in Ingenuity Pathway Analysis (www.ingenuity.com. Accessed 2012), MetaCore (GeneGo Inc.), PubMed (http://www.ncbi.nlm.nih.gov), nutritional metabolomics database for metabolites and proteins (http://wiki.nugo.org) and Human Metabolome database (http://www.hmdb.ca).

### Statistical Analysis

All data (except transcriptome) were analyzed for treatment differences using analysis of variance (ANOVA) taking possible cage-effects into account. If the ANOVA indicated an overall treatment effect, comparisons between treatment-means were performed. For all ANOVA models the ANOVA assumptions were checked by looking at the residuals from the ANOVA model. Data were log-transformed (natural logarithm) or rank-transformed if necessary. If the absolute value of a residual exceeded three times the residual standard deviation, the corresponding data point was removed from the data. Within subject changes over time were analyzed with paired Student’s T-test. For GC-MS, multiplex and eicosanoids data a Dunnett’s adjustment was used to correct for multiple comparisons between the high fat treatment and drug/lifestyle interventions. False discovery rate was used to correct for multiple testing (significance threshold FDR<0.05). The SAS statistical software package V9.1.3 (SAS institute Inc., North Carolina, USA) was used for univariate statistical analysis. Data are presented as mean ± SD, unless mentioned otherwise.

## Results

### Differential Effects of Drugs and Lifestyle Interventions on Risk Factors

Prior to intervention with pharmaceuticals or dietary lifestyle, male LDLr−/− mice were fed a high fat diet (HFD) for 9 weeks to establish risk factors of developing T2DM including mild obesity, hyperglycemia, hyperinsulinemia, hypercholesterolemia and hypertriglyceridemia (Table 1). In week 9, the mice were subjected to different interventions, for 7 weeks (Table 2). Body weight in HFD-fed control mice continued to increase, while chow control mice hardly gained weight. This effect was reflected by the epididymal fat weight, which was significantly increased in HFD mice compared to chow fed mice. The anti-diabetic drugs and anti-inflammatory compounds had no significant effects on body weight and epididymal fat. The LXR agonist T0901317 was the only drug of the lipid modulating compounds that significantly reduced body weight and epididymal fat compared to HFD at t = 16 weeks. Dietary lifestyle intervention (DLI), i.e. switching to a low-fat chow diet, resulted in a significant decrease in body weight and epididymal fat mass. In week 16, the DLI group was fully comparable to the control group which was kept on chow during the entire experimental period.

**Table 1:** Risk factors of developing T2DM induced by 9 weeks of HFD in LDLr−/− mice.

Parameter	T = 0	T = 9 weeks Chow	T = 9 weeks HFD
Body weight (g)	26.1±1.8	28.4±2.1	35.7±4.5*
Glucose (mM)	11.5±1.2	11.1±1.0	12.6±2.0*
Insulin (ng/mL)	0.6±0.5	0.4±0.3	2.7±2.1*
Cholesterol (mM)	5.3±0.7	7.9±2.0	13.7±4.4*
Triglycerides (mM)	1.2±0.2	1.3±0.3	2.2±1.1*

* P<0.05 compared to T = 0.

**Table 2:** The effect of the interventions on hallmarks and complications of T2DM.

group	bodyweight(g)	epididymalfat (mg)	glucose (mM)	insulin(ng/mL)	cholesterol(mM)	TG (mM)	intrahepaticTG(mmol/mg liver)	albumin/crea-tinine(µg/mg)	atherosclerosis(µm^2^)
**chow**	29.8±0.8	456±65	11.1±0.3	0.6±0.2	5.8±0.2	1.14±0.04	0.078±0.005	65.4±6.3	4308±850
**HFD 9w**	36.7±2.1	1459±320	12.5±0.7	2.9±0.8	13.8±2.1	1.86±0.35	0.115±0.033	89.1±7.0	4880±2355
**HFD 16w**	42.8±1.4	2280±150	15.0±0.5	4.3±0.8	20.3±1.4	3.08±0.46	0.183±0.024	186.5±8.1	23853±4464
**metformin**	38.3±2.7	2151±248	13.7±0.6#	3.3±0.9	14.8±1.8#	1.86±0.90	0.200±0.023	156.9±11.4	18960±5324
**rosiglitazone**	45.5±1.0	1818±113	10.6±0.2* ^,^ #	1.4±0.2	20.6±1.9	5.39±1.35	0.138±0.016	116.2±13.0*	58207±17472
**pioglitazone**	42.2±2.4	1987±232	12.2±0.7#	2.7±0.6	20.5±2.4	4.95±1.24	0.189±0.024	143.8±8.5	34295±8831
**sitagliptin**	42.9±2.2	2027±258	14.9±1.3	5.3±1.4	18.8±2.8	3.49±0.55	0.178±0.025	219.3±27.6	36222±14578
**glibenclamide**	38.9±2.4	1564±232	22.3±1.8* ^,^ #	2.3±0.5	14.6±1.1#	2.00±0.24	0.162±0.018	153.2±12.6	21470±5391
**fenofibrate**	37.6±1.7	1393±278	12.1±0.3	3.1±1.1	37.0±4.7* ^,^ #	9.03±2.19#	0.277±0.023	95.8±6.3*	63114±17575
**T0901317**	28.9±1.0*	458±42*	10.1±0.3*	1.1±0.3	30.0±2.0#	33.29±1.46* ^,^ #	0.261±0.045	112.4±6.6*	85614±13136
**atorvastatin**	42.2±2.2	2035±215	14.7±0.6	5.1±1.4	17.6±2.0	4.46±1.17	0.207±0.017	155.8±10.3	13040±2092
**salicylate**	39.2±2.8	1530±280	13.8±1.0	4.7±1.5	16.2±2.5#	2.61±0.56	0.221±0.043	156.4±12.3	30158±10678
**rofecoxib**	42.7±1.9	2251±181	14.0±0.5	4.9±1.2	16.3±1.5#	2.80±0.40	0.181±0.028	162.0±13.7	24502±6727
**DLI**	30.5±0.8*	488±67*	11.8±0.3*	1.0±0.3	6.4±0.3* ^,^ #	1.15±0.03*	0.070±0.004*	56.4±3.5*	16219±3600

Body weight, epidydimal adipose tissue weight, fasting plasma concentrations of glucose, insulin, cholesterol and triglycerides (TG) of the experimental groups are shown together with the intrahepatic TG concentrations, the urinary albumin/creatinine ratio and the atherosclerotic lesion area. Data represent the effects of drug intervention or dietary lifestyle intervention (DLI) at 16 weeks, *i.e.* the end of study. One group was sacrificed earlier and prior to the start of the interventions at 9 weeks (HFD 9w).

* P<0.05 compared to HFD 16w,

# P<0.05 for within subject change from 9 weeks to 16 weeks compared to HFD. Data are shown as mean ± SEM.

Continuous HFD feeding until week 16 also further increased fasting plasma glucose and insulin relative to week 9. Fasting glucose and insulin remained stable and low in chow control mice. The glucose-lowering drugs rosiglitazone and pioglitazone significantly decreased glucose, even below the starting level of the intervention. Metformin significantly attenuated the increase in plasma glucose compared to the increase observed in the HFD group. Sitagliptin had no significant effects and glibenclamide increased fasting glucose. Plasma insulin tended to decrease with rosiglitazone, and the other anti-diabetic drugs had no significant effects on insulin. Plasma glucose was also significantly reduced by the lipid modulator T0901317, fenofibrate fully prevented a further increase in glucose and atorvastatin attenuated hyperglycemia. These effects were achieved in absence of significant effects on plasma insulin (and T0901317 even tended to reduce insulin levels). The anti-inflammatory drugs also slightly attenuated a further increase in plasma glucose without affecting plasma insulin. DLI fully normalized glucose and insulin levels which were comparable to chow control.

Continuous HFD feeding also further increased plasma cholesterol while cholesterol levels remained constant and low on chow. Metformin and glibenclamide blocked the HFD-induced elevation of cholesterol almost completely, while the other glucose modulating drugs had no effect on plasma cholesterol. The lipid-modulating drug atorvastatin also tended to attenuate an increase in cholesterol whereas T0901317 and fenofibrate significantly increased plasma cholesterol. Both anti-inflammatory drugs attenuated the increase in plasma cholesterol. DLI was the only intervention that reduced plasma cholesterol beyond the starting level and comparable to chow control mice.

Similar to cholesterol, plasma triglycerides also increased gradually with continuous HFD feeding and chow reference mice remained low in plasma triglycerides. The glucose lowering agents had no significant effects on plasma triglycerides. Fenofibrate and T0901317 significantly increased triglycerides. Atorvastatin and the anti-inflammatory drugs did not affect plasma triglycerides while plasma triglyceride levels of the DLI group returned to chow control levels.

### Differential Effects of Drugs and Lifestyle Interventions on Diabetic Complications

We next analyzed hepatic triglyceride concentrations, albumin/creatinine ratio and atherosclerosis as measures of diabetic complications in liver, kidney and vessel wall (Table 2). HFD feeding resulted in liver steatosis which developed gradually over time and which was characterized by significantly higher intrahepatic triglyceride concentrations at the end of the study relative to control chow. None of the drug interventions had a significant effect on intrahepatic triglycerides. Rosiglitazone attenuated the HFD-induced development of steatosis slightly and fenofibrate and T0901317 even enhanced steatosis (all not significant). By contrast, DLI significantly reduced intrahepatic triglyceride levels and the livers of this group were comparable to chow control.

The urinary albumin/creatinine ratio, a marker of renal functioning and microvascular complications, increased gradually with HFD feeding and was elevated compared to chow. Micro-albuminuria was attenuated by rosiglitazone, while the other anti-diabetic drugs had no significant effect. Fenofibrate and T0901317 treated mice exhibited a significantly lower albumin/creatinine ratio than HFD controls, whereas atorvastatin and both anti-inflammatory drugs had no effect. In DLI mice, the albumin/creatinine ratio was fully normalized and similar to chow controls indicating complete reversal of micro-albuminuria.

In week 16, HFD treated mice developed early atherosclerosis and atherosclerotic plaques of moderate severity while chow control animals hardly displayed lesions. Atherosclerosis was not significantly affected by the drugs. Some drugs even tended to enhance atherosclerosis; the anti-diabetic drug rosiglitazone and the lipid-modulating drugs fenofibrate exhibited a greater lesion area than HFD controls but the effect did not reach statistical significance. Atorvastatin and DLI exhibited the smallest lesion area and mainly mild lesions (not significant).

### Effect of Interventions on Plasma Metabolome and Proteome

**Figure 1:**
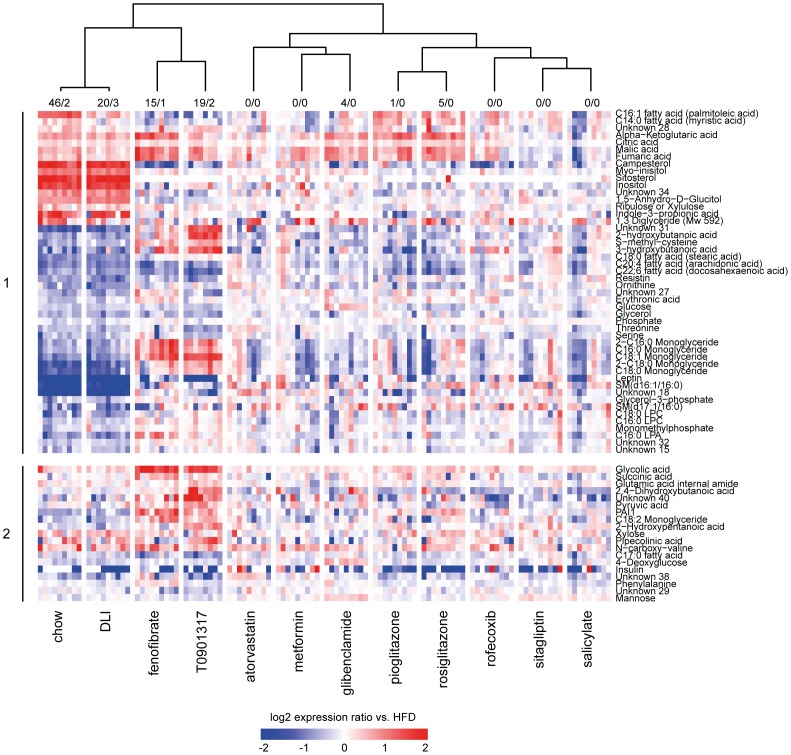
Effect of drug and dietary lifestyle interventions on protein and metabolite profiles in plasma. Shown are 67 proteins and metabolites with significantly different concentrations compared to HFD control group in at least one of the experimental conditions. The numbers of parameters (metabolites/proteins) with significantly different concentration compared to HFD control group in each experimental condition are provided above the heatmap. Log_2_ ratios of a particular group vs. mean of HFD control group are plotted in a heatmap. Each vertical lane within a treatment group represents a response of one mouse in that treatment group. The cluster tree (Pearson correlation, complete linkage) is based on average log_2_ ratios of a particular group vs. HFD. The upper part of the heatmap (1) represents proteins and metabolites that are significantly changed in the comparison chow control vs. HFD control group and defines thus changes that are associated with developing disease. The lower part of the heatmap (2) represents proteins and metabolites that are significantly changed in at least one of the interventions (i.e. dietary lifestyle (DLI) or one of the drug interventions) compared to HFD. The protein/metabolite profiles demonstrate a remarkable similarity between molecular signatures of chow control and DLI.

**Figure 2:**
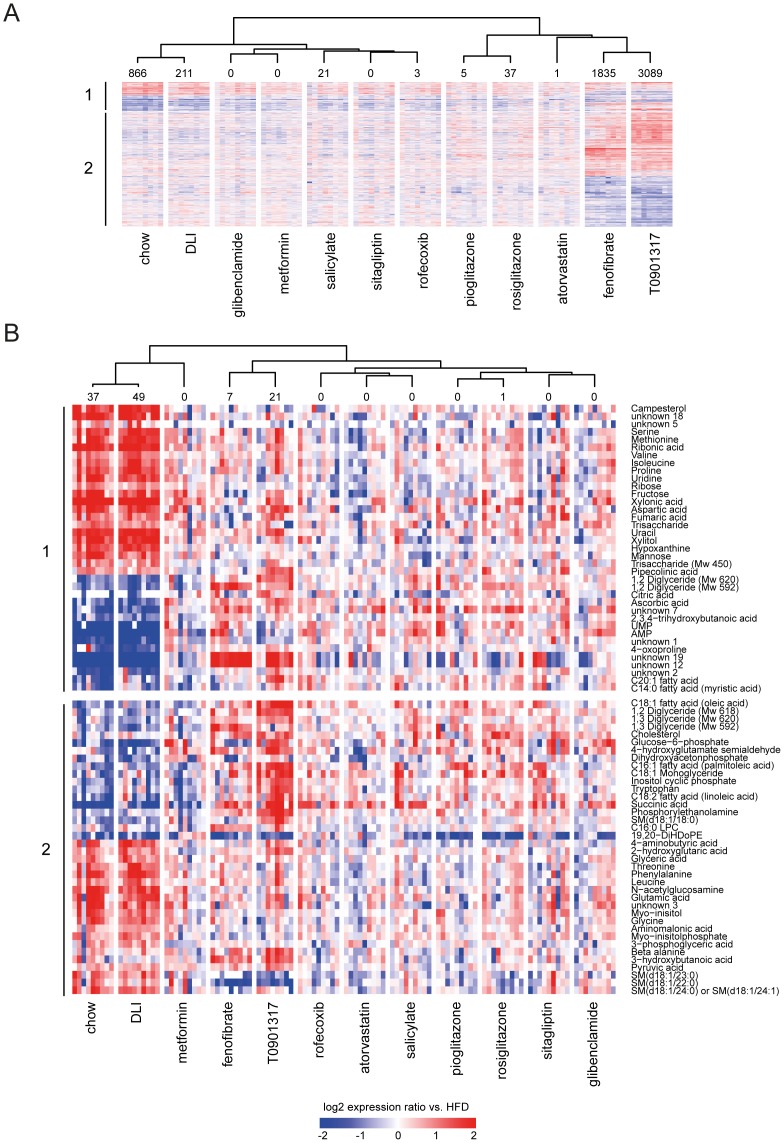
Effect of drug and dietary lifestyle interventions on hepatic transcriptome and metabolite concentration changes. The changes in expression (transcripts, A) or concentration (metabolites, B) that are significantly different in at least one of 12 experimental conditions compared to HFD group are plotted in a heatmap (log_2_ ratios vs. mean of HFD group). The number of significantly different transcripts or metabolites in each experimental condition is provided above the heatmap. The cluster tree (Pearson correlation, complete linkage) is based on average log_2_ ratios vs. HFD values per intervention group. The upper part of the heatmap (1) represents transcripts and metabolites that are significantly changed in chow control vs. HFD control and defines changes that are associated with developing disease. The lower part of the heatmap (2) represents transcripts/metabolites that are significantly changed in at least one of interventions (dietary lifestyle (DLI) or one of drug interventions) compared to HFD. The transcript/metabolite profiles demonstrate high similarity between molecular signatures of chow and DLI groups and pronounced effects of fenofibrate and T0901317. (A) The expression changes of 4286 transcripts that are significantly different in at least one of 12 experimental conditions, compared to HFD group. (B) The concentration changes of 75 metabolites that are significantly different in at least one of 12 experimental conditions, compared to HFD group.

To infer the effect of interventions on HFD-induced alterations of whole-body homeostasis, we profiled circulating plasma metabolites and proteins (153 molecules in total, Dataset S1). Compared to chow control, HFD treatment alone induced pronounced and significant changes in the plasma metabolome and proteome (Figure 1, Dataset S2). Changes include disease-related metabolites such as glucose, 2-hydroxybutanoic acid, 3-hydroxybutanoic acid, 1,5-anhydro-D-glucitol as well as endocrine factors such as leptin and resistin (P<0.05) which reflect the metabolic distortion of homeostasis in the circulation. HFD treatment also affected the plasma concentrations of monoglycerides and phospholipids, citric acid cycle intermediates, specific free fatty acids and glycerol while circulating inflammatory cytokines and branched chain amino acid concentrations remained unchanged (Figure 2, Figure S1).

Interventions with T0901317 and fenofibrate as well as DLI had a pronounced effect on the plasma metabolome and proteome profiles. The other drugs had no or minor effects on the plasma metabolome and proteome, suggesting a more local action of these drugs within their target tissues (Figure 1).

Hierarchical clustering analysis revealed that the DLI profile strongly resembled the plasma metabolite and protein profile of the chow control group, demonstrating that DLI reversed nearly all HFD-induced distortions. By contrast, T0901317 and fenofibrate reversed the profiles only partially: the plasma concentration of molecules related to citric acid cycle, amino acid metabolism, urea cycle, satiety as well as adipokines were similar to control group on chow, but ketone bodies and monoglycerides changed in an opposite way, possibly pointing to a further, yet partial, aggravation of specific metabolic distortions.

### Effect of Interventions on the Liver Transcriptome and Metabolome

To assess the impact of interventions on the homeostasis of a central metabolic organ, we analyzed the liver by combined transcriptomics and metabolomics (Figure 2, Dataset S2). The lipid-modulating drugs T0901317 and fenofibrate (which target the liver to activate the transcription factors LXR and PPARα, respectively) showed pronounced effects on the hepatic transcriptome and metabolome. Rosiglitazone, pioglitazone and rofecoxib affected the liver metabolome and transcriptome profiles to a much lesser extent, and other drugs hardly had an effect. By contrast, the liver transcriptome and metabolome profiles of the DLI and chow control groups were remarkably alike. This is consistent with the plasma profiling data and indicates that DLI reversed metabolic distortions caused by HFD feeding.

### Identification of Hepatic Processes Induced by HFD and Effect of Interventions

**Figure 3:**
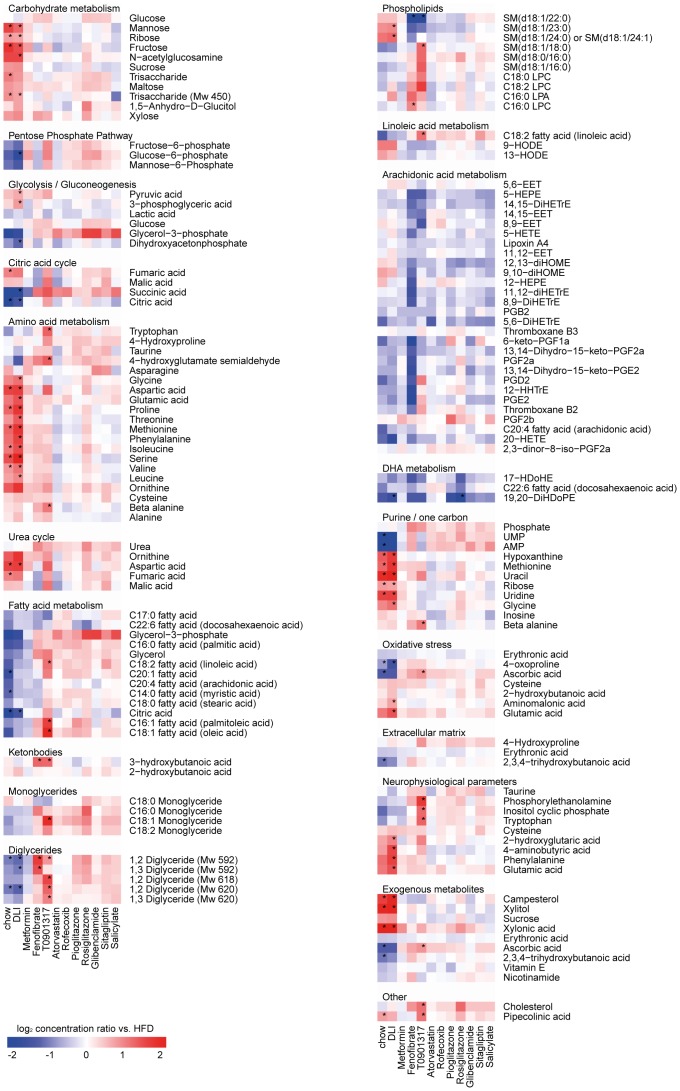
Identification of key biological processes based on significant changes in hepatic metabolites. The significantly changed hepatic metabolites in at least one of 12 conditions subdivided in biological processes (molecules with an unknown identity were excluded). Each lane represents the response of a treatment group expressed as mean log_2_ ratio vs. HFD group. Red indicates higher and blue indicates lower concentrations of the hepatic metabolite after treatment compared to HFD group. * indicates significantly changed metabolite concentrations after treatment as compared to HFD group with a p-value<0.05 after FDR correction.

To identify the biological processes affected by HFD, liver transcript and metabolite datasets were analyzed for functional categories. Only interventions with significant changes in the hepatic transcriptome and metabolite profiles were included in the analysis. Compared to chow control, HFD affected the metabolism of carbohydrates, lipids, fatty acids and amino acids (Figure 3, Figure S2). Also, alterations in growth and/or tissue remodeling processes and changes in concentrations of intermediates of purine metabolism were detected. Furthermore, prominent changes in (metabolic) stress and immune/inflammatory responses, oxido-reductive processes and circadian rhythm were observed.

**Figure 4:**
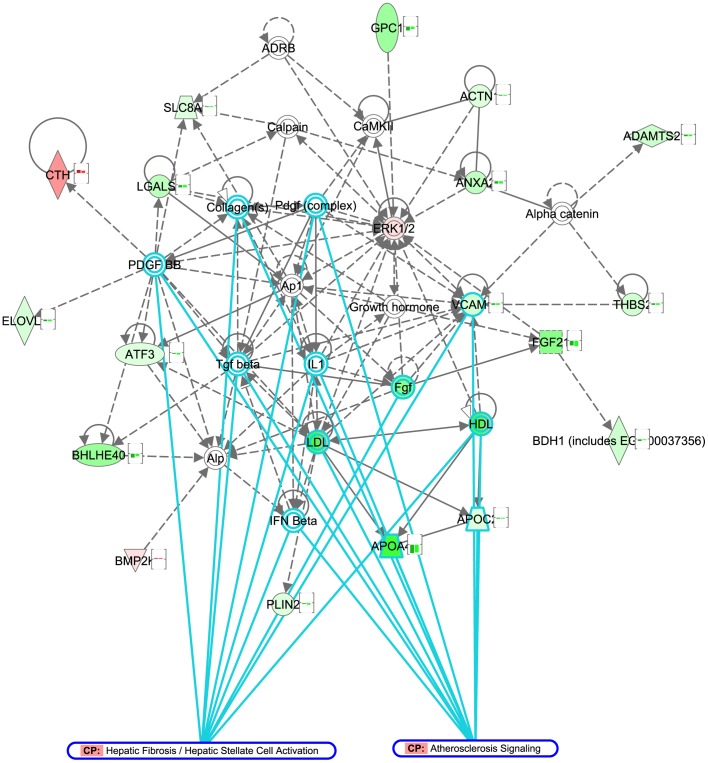
Molecular network of genes related to atherosclerosis signaling and hepatic fibrosis signaling pathway. Genes differentially expressed in dietary lifestyle intervention (DLI), compared to HFD were subjected to network analysis (Ingenuity Pathway Analysis). The network of genes associated with processes “Cellular Growth and Proliferation”, “Connective Tissue Development and Function” and “Hepatic System Development and Function” (network score 32) is represented in the figure. Genes or gene products are represented as nodes, and the biological relationships between two nodes are represented as edges (lines). The nodes of the network are colored according to log_2_ gene expression changes in the DLI vs. HFD comparison (red: upregulation, green: downregulation). The bar graph associated with each node represents log_2_ expression changes in chow (1st bar) and DLI groups (2nd bar) vs. HFD group, highlighting that all represented genes change in equivalent direction in chow and DLI conditions. The function “Overlay: Canonical Pathway” was used to highlight network genes associated with “atherosclerosis signaling” (11 genes, top enriched pathway) and “hepatic fibrosis/hepatic stellate cell activation” (9 genes, 3rd enriched pathway). All genes associated with these pathways, as well as majority of genes in the network are downregulated in DLI group, indicating withdrawal of pathogenic signals upon dietary lifestyle intervention.

DLI reversed most of these processes to the level of chow control. More specifically, combined pathway and metabolite analysis revealed that DLI normalized the glycolysis/gluconeogenesis pathways (downregulation of *GCK* ***,*** *ALDOB, GAPDH, PKLR, GPD2 and LDHA* and decreased glucose-6-phosphate concentrations), which may explain the improvement of the glycemic status found with DLI. Similarly, the beneficial effect of DLI on plasma cholesterol and triglyceride levels coincided with hepatic shutdown of unsaturated fatty acid biosynthesis **(**downregulation of *FADS1, FADS2* and *ELOVL2*), increased lipid degradation/oxidation (upregulation of *CYP3A11, CYP1A2, CYP26A1, CPS1, CYP27A1* and *GM2A*) and downregulation of lipid storage and genes coding for lipoproteins (*PLIN2, APOA4* and *APOC2*). Other hepatic pathways that may be involved in the observed restoration of a “healthy” phenotype by DLI are those required for restoring the balance in energy homeostasis (upregulation of cytochrome P450 family, enrichment of mitochondrial proteins) as well as the shutdown of pathogenic mechanisms (immune response, cell cycle, apoptosis and tissue remodeling processes). In line with the latter, the majority of genes related to atherosclerosis signaling and hepatic fibrosis were downregulated by DLI (Figure 4).

**Figure 5:**
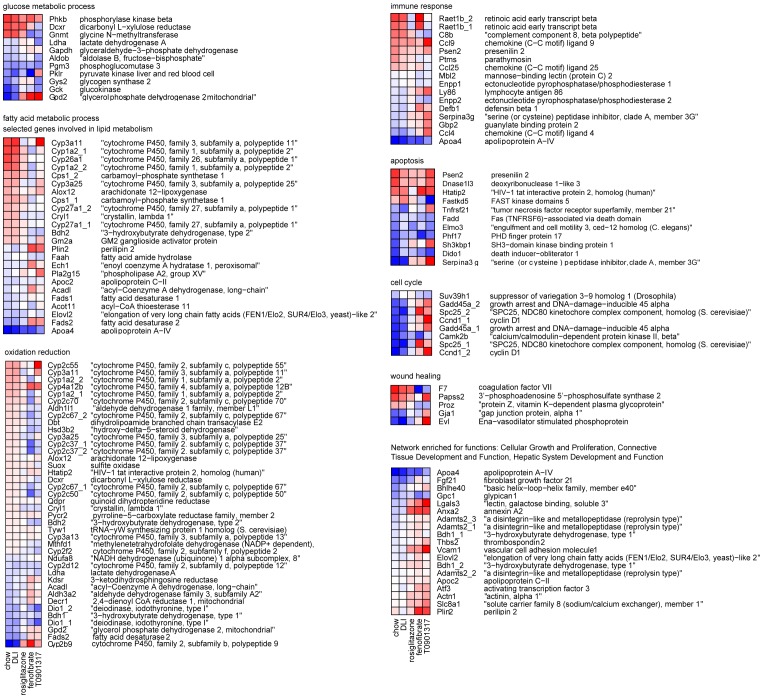
The effect of rosiglitazone, fenofibrate and T090131 on key hepatic processes required for the reversal of HFD-induced disorders. The Gene Ontology biological processes “glucose metabolic process”, “fatty acid metabolic process”, “oxidation reduction”, “immune response”, “apoptosis”, “cell cycle” and “wound healing” were selected for a detailed analysis to investigate whether the hepatic transcriptome changes induced by the drugs with marked hepatic effects (fenofibrate, T090131 and rosiglitazone) are in line with the changes induced by DLI, i.e. a “return to healthy” profile. The heatmaps show the mean log_2_ expression ratio of each treatment vs. HFD group for genes involved in these processes (red: upregulation, blue: downregulation). Both fenofibrate and T090131 show many additionally and oppositely changing genes compared to DLI and/or chow group. Rosiglitazone has minor effects all of which were in line with the changes in chow/DLI groups.

Since hepatic pathways affected by DLI are likely relevant for reversing the disease, we investigated whether the hepatic transcriptome changes induced by the drugs with pronounced liver effects (fenofibrate, T090131 and rosiglitazone) would be in the same direction as the effects of DLI. To do so, the Gene Ontology biological processes “glucose metabolic process”, “fatty acid metabolic process”, “oxidation reduction”, “immune response”, “apoptosis”, “cell cycle” and “wound healing” were selected for a detailed analysis (Figure 5, Table S1). Both fenofibrate and T090131 had a major effect on these processes but their transcriptional effects were partly opposite to DLI. Rosiglitazone, by contrast, had less pronounced effects than the other two drugs, but all transcriptional changes were in the same direction as DLI.

## Discussion

It is well-established that intensive glucose control can slow the progression of micro-vascular complications of T2DM [22], [23], but the overall success rate of this approach to reduce other complications, in particular cardiovascular events, is rather disappointing [5], [24]. Many of the drugs analyzed in this study targeted blood glucose efficiently which is in accordance with their presumed mode of action and effect in patients [25]–[27]. However, drug interventions did not or only partially resolve other risk factors and T2DM-associated complications. Only rosiglitazone, fenofibrate and the LXR-agonist T0901317 improved micro-albuminuria which is in accordance with the benefit reported for rosiglitazone and fenofibrate in human studies investigating microvascular endpoints [28]–[30]. In contrast to the drug interventions, removal of the dietary overload by switching to a low-fat chow diet (DLI group) demonstrates the ability to re-establish a “healthy” phenotype on most investigated levels, i.e. traditional risk factors of T2DM (fasting glucose, fasting insulin, body weight and adiposity), cardiovascular risk factors (cholesterol, triglycerides) and diabetic complications in liver (steatosis) and kidney (microalbuminuria). Also, intra-hepatic metabolites and gene expression levels as well as circulating metabolites and proteins normalized within only 7 weeks of DLI indicating a rapid restoration of homeostasis in organs and systemically.

Most of the anti-diabetic drugs tested herein prevented or attenuated the HFD-induced increase in fasting glucose which is in line with their expected therapeutic effect. Long-term chronic treatment with glibenclamide, however, resulted in an increase in fasting plasma glucose. The continuous treatment with glibenclamide in this study may have caused secondary failure (i.e. relapse of hyperglycemia) due to an inhibition of beta-cell K-ATP channels as it has been reported earlier for this drug in rodent studies [31]. Such effects were also observed in patients treated with sulfonylurea for prolonged time [32]. In general however it is not possible to draw conclusions on humans who have complex pathogenesis of the disease and treatment strategies depending on duration of the disease and comorbidities.

Most of the pharmaceuticals employed had a beneficial effect on blood glucose but only little effect on T2DM-associated complications. Rosiglitazone, fenofibrate and the LXR-agonist T0901317 improved the albumin/creatinine ratio, but the same compounds tended to promote the development of atherosclerosis. Three out of nine rosiglitazone treated LDLr−/− mice showed pronounced atherosclerosis with severe lesions. Indeed, adverse cardiovascular effects have been reported in some patients and are indeed safety concern for this drug [33]–[35]. We observed adverse cardiovascular effects of rosiglitazone in a setting of HFD-induced T2DM. Others reported an attenuating effect of this drug on atherosclerosis using the same strain of mice [36]. However, different diets were used and the atheroprotective effect was observed with a cholesterol-containing atherogenic diet which induces a different spectrum of risk factors. This shows that it is extremely difficult to extrapolate an effect of a particular drug measured under atherogenic conditions to a diabetic conditions. Different diets may induce different disease pathways within the vasculature and the factors which participate in the disease process may differ.

T0901317 treatment increased plasma triglycerides and cholesterol levels as reported previously for this drug by us and others [37]–[39]. Under conditions of experimental T2DM, T0901317 treated mice tended to develop more atherosclerosis than the HFD control group. In absence of conditions of T2DM, T0901317 treatment diminished the lesion load [38]. A recent study, performed in LDLr−/− mice, shows differential pro- and anti-atherogenic effects of T0901317 depending on the vascular bed analyzed [40] which may explain the discrepancy. Another explanation may be related to the different diets used (and thus cardiovascular risk factors induced) as outlined above for rosiglitazone. Indeed, studies showing atheroprotective effects of T09031317 employed atherogenic diets which contain high concentrations of cholesterol [37] or cholesterol/cholate [38]. The HFD used in herein was not supplemented with cholesterol. Because LXR activators are stimulating cholesterol efflux (as part of their atheroprotective effect) it is likely that differences in cholesterol content of diets may skew a disease model towards a particular phenotype in which cholesterol trafficking plays a greater role.

An important finding of this study is that the disease process is reversible and that risk factors and associated early pathologies (micro-albuminuria, hepatosteatosis) can be resolved. It requires further analyses to investigate whether the disease can be reversed at all stages or whether there is a ‘point of no return’. The fact that atherosclerosis in the DLI group was not resolved within a period of 7 weeks suggests that such a point may exist for macrovascular complications. On the other hand, it is also possible that restoration of macrovascular damage is a relatively slow process which requires either more time to regress as reported [41], or pharmacotherapy [37], [42].

Consistent with the pronounced effect of DLI herein, early intervention with intensive lifestyle reduces the cardiovascular risk and improves the glucose tolerance profile in subjects at risk for T2DM [43]. Also, a meta-analysis of thirteen human studies investigating the effect of lifestyle interventions points to a reversion of early diabetic phenotype with lifestyle [44] which is consistent with our findings. Data of this meta-analysis however do not allow to assess whether these benefits are sustained and will translate into longer term prevention of cardiovascular disease.

Multiple risk factors can contribute to T2DM development in an individual patient, and the nature of the underlying disease processes can vary between patients. Targeting single targets or single risk factors – for instance using a ‘one drug, one target approach’ - is likely an ineffective strategy to resolve the entire complexity of the disease phenotype. Restoring the balance in one physiological process may lead to re-routing of the problem to other part(s) of the system. Combinations of drug therapies may cover multiple disease-associated manifestations, but have the increased likelihood of undesirable side effects as a tradeoff [45]. Lifestyle intervention does not act on the disease symptoms but rather withdraws the cause of disease (i.e. metabolic pressure). Consequently, the problem is tackled at its origin and therefore has a realistic potential to systemically reverse the disease. It is of importance to gain further insight into the molecular mechanisms of DLI in order to harmonize lifestyle and pharmacotherapy optimally.

Besides the evidently relevant processes, such as glucose, lipid/fatty-acid metabolism and inflammation, the present study identified processes related to the redox status of the cell, cell cycle/apoptosis and tissue remodeling as vital for re-establishing the health phenotype by DLI. This supports emerging therapeutic approaches for metabolic disease such as restoring endoplasmic reticulum function and stress [46].

Taken together, the presented findings provide insight into the limitations of current T2DM treatment regimens and may open new avenues for novel therapeutic paradigms based on a systems approach. DLI has the potential to fully resolve HFD induces metabolic distortions in the liver and systemically, and stops the progression of T2DM and important complications associated with it. Insight from DLI may be used for development of rational drug (combination) therapies that mimic the beneficial effects of DLI.

## Supporting Information

Figure S1 **Biological processes represented among significantly changed plasma metabolites and proteins.** The significantly changed plasma metabolites and proteins in at least one of 12 conditions are subdivided in biological processes (molecules with an unknown identity were excluded). Each lane represents the response of a treatment group expressed as mean log2 ratios vs. HFD group. Red indicates higher and blue indicates lower concentrations of the plasma molecule after treatment compared to HFD group. *indicates significantly changed molecule concentrations after treatment as compared to HFD group with a p-value<0.05 after FDR correction.
(PDF)

Figure S2 **Biological processes affected by drug and dietary lifestyle interventions on a transcriptome level.** Representative Gene Ontology Biological Process categories overrepresented among differentially expressed transcripts in dietary lifestyle (DLI) and drug intervention groups (vs. HFD), and their corresponding p-values (heatmap). The clustering tree highlights a similarity between process-enrichment profiles of chow control and DLI groups and between fenofibrate and T0901317 groups.
(PDF)

Table S1 **The effect of rosiglitazone, fenofibrate and T090131 on key hepatic processes required for the reversal of HFD-induced disorders.** Gene Ontology biological processes “glucose metabolic process”, “fatty acid metabolic process”, “oxidation reduction”, “immune response”, “apoptosis”, “cell cycle” and “wound healing” were selected for a detailed analysis to compare to the changes induced by DLI, i.e. a “return to healthy” profile. The number of genes in these processes that are regulated in the same or opposite manner between T090131, fenofibrate or rosiglitazone group and DLI group are shown.
(PDF)

Dataset S1 **Overview of metabolites and proteins analyzed in plasma and liver.** GCMS metabolic profiling platform nr 1, multiplex proteome analyses platform nr 2 and Eicosanoid LCMS analyses platform nr 3.
(XLS)

Dataset S2 **Significantly changed plasma and hepatic molecules in the different treatments (week 16) compared to 16 weeks of HFD.** FDR significance: 0 = > not significant after FDR correction. FDR significance: 1 = > significant after FDR correction. Abbreviations used: DMCs: Differentially changed Metabolite Concentrations, DPCs: Differentially changed Protein Concentrations. DEPs: Differentially Expressed Probes, FC: fold change, FDR: false discovery rate. LDLR: low density lipoprotein receptor-deficient mice, DLI: Dietary Lifestyle Intervention.
(XLS)
