# Supplementary material for: Differential Effects of Drug Interventions and Dietary Lifestyle in Developing Type 2 Diabetes and Complications: A Systems Biology Analysis in LDLr−/− Mice
Source: PLoS One. 2013 Feb 15;8(2):e56122. doi: 10.1371/journal.pone.0056122 (PMC3574110; doi:10.1371/journal.pone.0056122)
Supplement: Figure S1 — Biological processes represented among significantly changed plasma metabolites and proteins. The significantly changed plasma metabolites and proteins in at least one of 12 conditions are subdivided in biological processes (molecules with an unknown identity were excluded). Each lane represents the response of a treatment group expressed as mean log2 ratios vs. HFD group. Red indicates higher and blue indicates lower concentrations of the plasma molecule after treatment compared to HFD group. *indicates significantly changed molecule concentrations after treatment as compared to HFD group with a p-value<0.05 after FDR correction. (PDF) [file pone.0056122.s001.pdf]

Supporting Information Fig 1

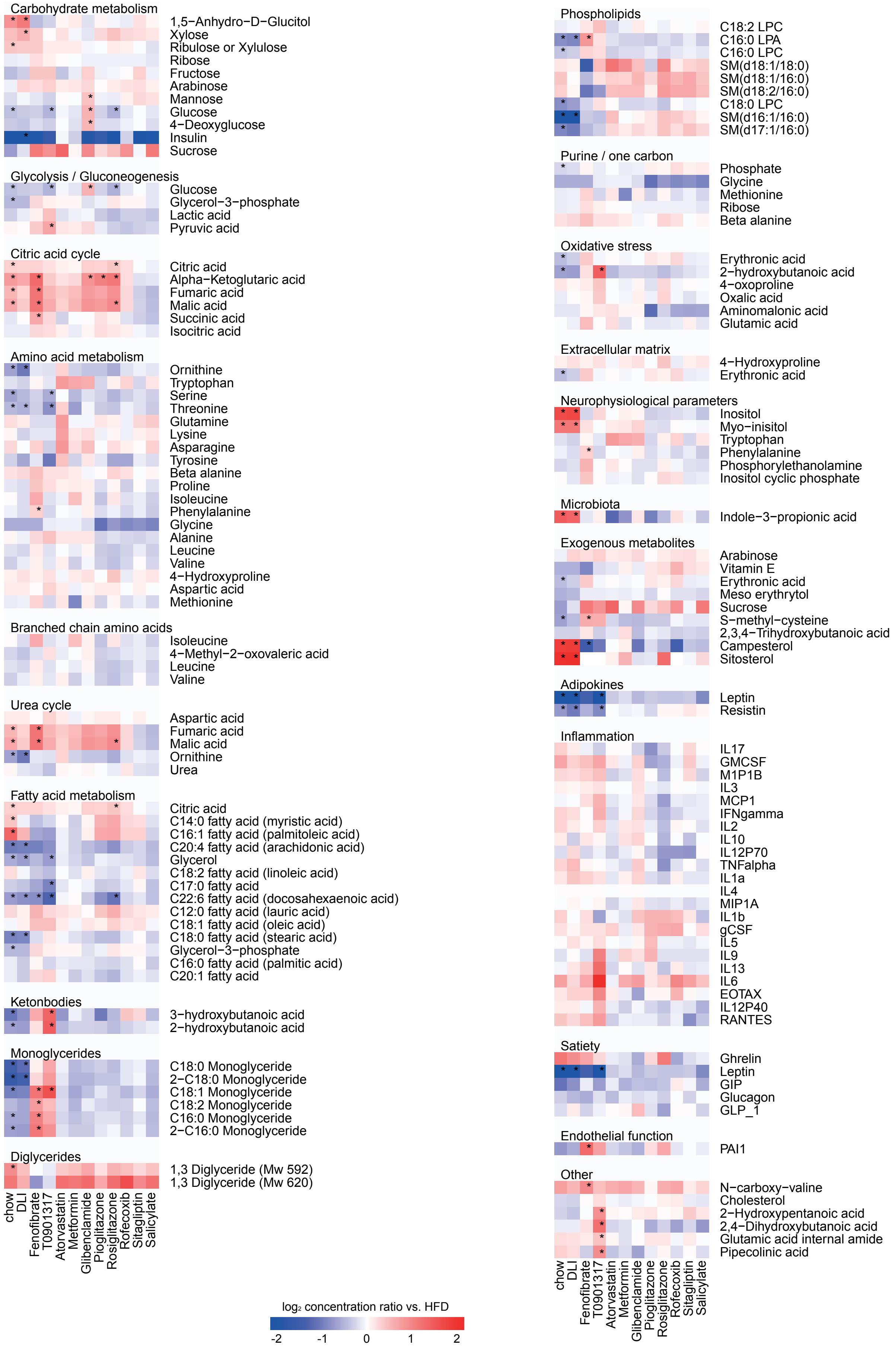

Supporting Information Fig 1. Biological processes represented among significantly changed plasma metabolites and proteins

The significantly changed plasma metabolites and proteins in at least one of 12 conditions are subdivided in biological processes (molecules with an unknown identity were excluded). Each lane represents the response of a treatment group expressed as mean log2 ratios vs. HFD group. Red indicates higher and blue indicates lower concentrations of the plasma molecule after treatment compared to HFD group. * indicates significantly changed molecule concentrations after treatment as compared to HFD group with a p-value < 0.05 after FDR correction.
