# Supplementary figures and images for: Differential Effects of Drug Interventions and Dietary Lifestyle in Developing Type 2 Diabetes and Complications: A Systems Biology Analysis in LDLr−/− Mice
Source: PLoS One. 2013 Feb 15;8(2):e56122. doi: 10.1371/journal.pone.0056122 (PMC3574110; doi:10.1371/journal.pone.0056122)

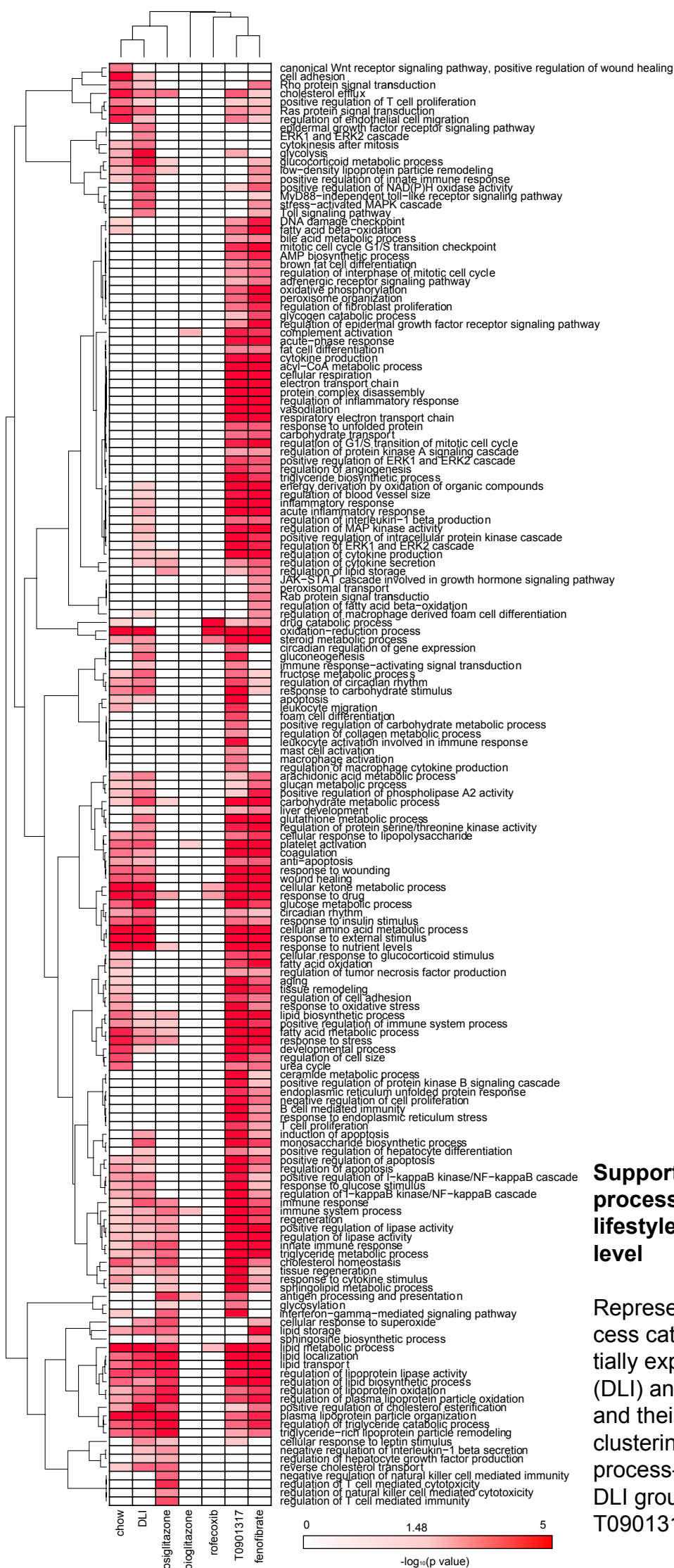

Supplement: Figure S2 — Biological processes affected by drug and dietary lifestyle interventions on a transcriptome level. Representative Gene Ontology Biological Process categories overrepresented among differentially expressed transcripts in dietary lifestyle (DLI) and drug intervention groups (vs. HFD), and their corresponding p-values (heatmap). The clustering tree highlights a similarity between process-enrichment profiles of chow control and DLI groups and between fenofibrate and T0901317 groups. (PDF) [file pone.0056122.s002.pdf]
