# Supplementary material for: Differential Effects of Drug Interventions and Dietary Lifestyle in Developing Type 2 Diabetes and Complications: A Systems Biology Analysis in LDLr−/− Mice
Source: PLoS One. 2013 Feb 15;8(2):e56122. doi: 10.1371/journal.pone.0056122 (PMC3574110; doi:10.1371/journal.pone.0056122)
Supplement: Table S1 — The effect of rosiglitazone, fenofibrate and T090131 on key hepatic processes required for the reversal of HFD-induced disorders. Gene Ontology biological processes “glucose metabolic process”, “fatty acid metabolic process”, “oxidation reduction”, “immune response”, “apoptosis”, “cell cycle” and “wound healing” were selected for a detailed analysis to compare to the changes induced by DLI, i.e. a “return to healthy” profile. The number of genes in these processes that are regulated in the same or opposite manner between T090131, fenofibrate or rosiglitazone group and DLI group are shown. (PDF) [file pone.0056122.s003.pdf]

Supporting Information Table 1

GO Biological Process term	Rosiglitazone			Fenofibrate			T0901317		
	equivalent*	opposite**	additional***	equivalent	opposite	additional	equivalent	opposite	additional
glucose metabolic process	1	0	0	3	2	16	5	3	31
fatty acid metabolic process	0	0	0	2	4	58	2	4	59
oxidation reduction	0	0	2	8	19	157	15	15	224
immune response	2	0	6	3	0	61	6	6	134
apoptosis	0	0	1	3	0	39	7	4	79
cell cycle	0	0	0	2	2	37	1	4	67
wound healing	0	0	0	0	2	24	1	3	42

*, ** Number of differentially expressed probes that change in equivalent (*) or opposite (**) direction to chow and/or DLI group vs. HFD

*** Number of differentially expressed probes from GO category that are changed in drug group, but not in chow and/or DLI group vs. HFD

Supporting Information Table 1. The effect of rosiglitazone, fenofibrate and T090131 on key hepatic processes required for the reversal of HFD-induced disorders

Gene Ontology biological processes “glucose metabolic process”, “fatty acid metabolic process”, “oxidation reduction”, “immune response”, “apoptosis”, “cell cycle” and “wound healing” were selected for a detailed analysis to compare to the changes induced by DLI, i.e. a “return to healthy” profile. The number of genes in these processes that are regulated in the same or opposite manner between T090131, fenofibrate or rosiglitazone group and DLI group are shown.
